# Primary T‐cell‐based delivery platform for in vivo synthesis of engineered proteins

**DOI:** 10.1002/btm2.10605

**Published:** 2023-10-07

**Authors:** Harikrishnan Radhakrishnan, Sherri L. Newmyer, Marvin A. Ssemadaali, Harold S. Javitz, Parijat Bhatnagar

**Affiliations:** ^1^ Biosciences Division SRI International Menlo Park California USA; ^2^ Education Division SRI International Menlo Park California USA

**Keywords:** CAR T cells, cell engineering, cell manufacturing, cell‐based drug delivery, synthetic biology

## Abstract

Primary T cell has been transformed into a *cell‐based delivery platform* that synthesizes complex biologics at the disease site with spatiotemporal resolution. This broadly applicable technology can circumvent toxicities due to systemic administration of biologics that necessitates the use of high doses and may diffuse to the healthy tissues. Its clinical translation, however, has been impeded by manufacturing bottlenecks. In this work, a range of process parameters were investigated for increasing the production yield of the primary T cells engineered for delivery function. Compared to the common spinoculation‐based method, the transduction yield was enhanced ~2.5‐fold by restricting the transduction reaction volume for maximizing the lentivector‐to‐T‐cell contact. Cell density and cytokines used in the expansion process were adjusted to achieve >100‐fold expansion of the T‐cell‐based delivery platform in 14 days, and the function of these cells was validated in vivo using intraperitoneally implanted tumor cells. The primary T‐cell‐based delivery platform has human applications because it can be scaled and administrated to express a broad range of therapeutic proteins (e.g., cytokines, interferons, enzymes, agonists, and antagonists) at the disease site, obviating the need for systemic delivery of large doses of these proteins.

**Translational Impact Statement**

Primary T cell has been transformed into a platform for synthesizing complex biologics directly at the disease site with precise timing and location. Unlike the current status‐quo of first‐order drug delivery systems that present systemic biodistribution and can affect healthy tissues, this technology may be used to synthesize engineered proteins so as to exert desired therapeutic effects by autocrine or paracrine signaling only at the disease site without affecting healthy tissues. In vivo experiments confirmed the successful synthesis of functional proteins by the engineered cells.

## 1 INTRODUCTION

The current approach for treating many diseases that progress in local tissue microenvironments (e.g., cancers, viral infections, autoimmune disorders) is based on drug doses normalized to body weight and surface area^1^ but does not account for interpatient variability. Continuous monitoring of the disease state following each dose is not practical.^2^, ^3^ Overdoses cause morbidity, and suboptimal dosing leads to drug resistance. This misalignment between the disease state and the administered dose^4^, ^5^ has led to serious adverse events.^6^, ^7^ To address this issue, we have developed a cell‐based delivery platform that can assess the disease burden and express proportionate amounts of biologic drugs in a site‐specific manner. T cells are particularly useful in this setting because they chemotactically extravasate to the disease site with cellular resolution and, on engaging the target cells with molecular specificity, activate to exert their effector function in an on/off manner.^8^, ^9^, ^10^, ^11^, ^12^ The T cell can therefore synthesize the therapeutic payload only within the disease microenvironment, offering the potential to spare healthy cells from undesired side effects.

To demonstrate the feasibility of this cell‐based delivery system, we engineered a synthetic pathway in an acute T‐lymphoblastic leukemia cell line (Jurkat T cell).^13^ The synthetic pathway incorporates a chimeric antigen receptor (CAR) that, upon engaging the target cell in an antigen‐specific manner, induces the expression of desired protein through the nuclear factor of activated T cells response element (NFAT‐RE)‐signaling pathway, and does so proportionate to the number of target cells.^13^ We verified that the synthetic pathway is functional in NK‐92MI,^14^ a cell line that is currently being tested as an off‐the‐shelf cell therapy in human clinical trials.^15^, ^16^ Translating this synthetic pathway into primary T cells is vital for the safe and effective application of this cell‐based delivery technology in humans. However, the manufacturing challenges (e.g., low transduction and expansion yield of the primary T cells and the negative impact of these processes on the function of the engineered cells) continue to present a formidable barrier to clinical adoption.^17^, ^18^, ^19^, ^20^ The larger size of the genetic payload, relative to CAR T cells, additionally complicates an already difficult process of generating a full dose for Phase I/II clinical trials.

**FIGURE 1:**
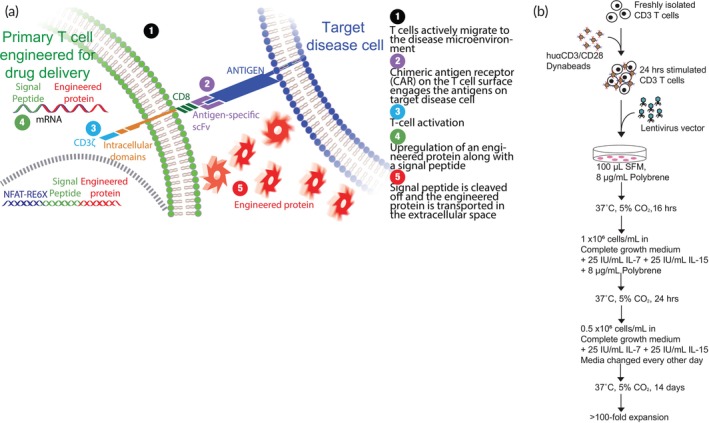
Primary T‐cell‐based delivery platform. (a) Schematic of the engineered primary T‐cell‐based delivery platform. (b) Schematic of the process for manufacturing the engineered primary T‐cell‐based delivery platform. SFM, serum‐free media, αhu: anti‐human.

Toward our overall goal of developing affordable cell therapy solutions, we previously reported a scalable process for manufacturing lentiviral vector for engineering primary T cells into a cell‐based delivery system.^21^ In this work, using a CAR with specificity for folate receptor alpha (FRα) and a reporter enzyme to represent the desired biologic, we described a simple process to efficiently scale the primary T‐cell‐based delivery platform (Figure 1). As recently demonstrated by us,^13^, ^14^, ^22^, ^23^, ^24^, ^25^ however, the DNA template of the antigen‐sensing scFv domain and the reporter enzyme can be exchanged to redirect the specificity of this platform to another clinically relevant antigen and express a protein for sensing^23^, ^25^ or therapeutic^22^, ^24^ function.

## 2 RESULTS

### 2.1 Investigation of parameters affecting transduction of primary T cells with lentivectors

**FIGURE 2:**
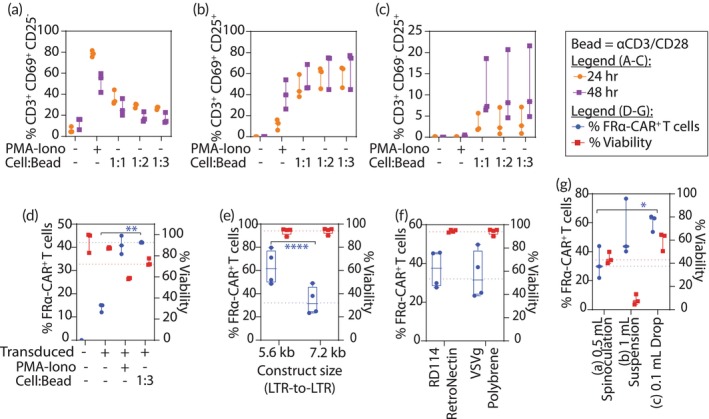
Factors affecting transduction of primary T cells with lentivectors. (a–c) T‐cell activation marker (CD25, CD69) expression in CD3 T cells (*n* = 3 donors) was assessed by flow cytometry at 24‐ or 48‐h after stimulation by chemicals (Phorbol 12‐myristate 13‐acetate [30 nM] and ionomycin [1 μM], PMA/Io) or by different cell‐to‐bead (Dynabeads loaded with antihuman CD3 and antihuman CD28) ratios. Strategy for evaluating CD3 T cell activation is presented in Figure S1. Different stages of T‐cell activation are shown in (a) early activation (CD69^+^CD25^−^), (b) peak activation (CD69^+^CD25^+^), and (c) late activation (CD69^−^CD25^+^). (d) FRα‐CAR expression (% FRα‐CAR^+^ T cells on left y‐axis) and T‐cell viability (% Viability on right y‐axis) was assessed by flow cytometry after transducing stimulated and unstimulated primary T cells. (e–g) FRα‐CAR expression (% FRα‐CAR^+^ T cells on left y‐axis) and T‐cell viability (% Viability on right y‐axis) was assessed by flow cytometry after varying factors affecting transduction (e) size of the genetic payload (chimeric antigen receptor [CAR] only, 5.6 kb vs. T‐cell‐based delivery system comprising of CAR and nuclear factor of activated T cells response element [NFAT‐RE] inducible transgene, 7.2 kb), see Figure S2 for schematics, (f) lentivector pseudotype (RD114 vs. VSV‐g), and (g) lentivector transduction methods (Method a: spinoculation at 800*g* in 0.5 ml for 1.5 and 14.5 h in cell culture incubator; Method b: 1.0 ml reaction volume for 16 h in cell culture incubator; Method c: 0.1 ml reaction volume for 16 h in cell culture incubator). Transduction efficiency was determined after 5 days. All results are represented as mean ± SD. Statistical analysis and *p* values for (d) and (g) were determined by one‐way ANOVA and Tukey's multiple comparison test. Statistical analysis and *p* values for (e) were determined by Student's *t* test, two‐tailed. *p < 0.05, **p < 0.01, ***p < 0.001 and ****p < 0.0001.

Figure 2 shows the effect of various parameters on the lentivector transduction of primary T cells, all of which can improve manufacturing efficiency of the cell‐based delivery platform. Figure 2a–c demonstrates the effect of the cell‐to‐bead ratio on early (CD69^+^CD25^−^) (Figure 2a), peak (CD69^+^CD25^+^) (Figure 2b), and late (CD69^−^CD25^+^) (Figure 2c) activation of CD3 T cells (see Figure S1 for the gating strategy using a representative fluorescence‐activated cell sorting (FACS) plot). Figure 2b shows that 60% of CD3 T cells progress to peak activation (CD69^+^CD25^+^) within 24 h after stimulation by beads at a cell‐to‐bead ratio of 1:3. As such, we adopted this ratio in our manufacturing process. Figure 2d compares the transduction efficiency of T cells activated by beads at 1:3 to chemically activated T cells (Phorbol 12‐myristate 13‐acetate and ionomycin, PMA/Io). Although the transduction efficiency was similar in magnitude (42% vs. 40%, *n* = 3), the yield for producing engineered FRα‐CAR^+^ T cell was higher when activated with beads compared to PMA/Io (75% vs. 60%, *n* = 3). The transduction efficiency was ~15% in nonactivated T cells.

Figure 2e–g compares other parameters that affect the transduction efficiency of primary T cells. We assessed improvement as the percentage of modified primary T cells (% FRα‐CAR^+^ T cells, left y‐axis) and the number of live primary T cells (% viability, right y‐axis) in the culture 5 days after transduction.

Figure 2e shows the effect of the size (LTR‐to‐LTR) of genetic payload, 7.2 kb (for the T‐cell‐based delivery system comprising the CAR and NFAT‐RE inducible transgene)^13^ versus 5.6 kb (CAR only), using the same plasmid vector (Figure S2a,b). We did not observe any difference in cell viability, but the resulting transduction yield for transducing primary T cells with the 7.2 kb genetic payload was ~50% less compared to that of the 5.6 kb genetic payload (62.85 ± 14% vs. 33.8 ± 15%, *n* = 4 donors). This is consistent with a previous report on the decreased efficiency of lentivector transduction with an increase in transgene length.^26^

To address the reduced yield in generation of the primary T‐cell‐based delivery platform, we compared the two pseudotyped lentivirus vectors for transducing T cells: (i) VSV‐g envelope protein from vesicular stomatitis virus and (ii) RD114 envelope protein from infectious feline endogenous retrovirus.^27^ The VSV‐g envelope protein is widely accepted for engineering T cells, and RD114 has been reported to improve efficiency in engineering CD34 hematopoietic cells^28^ and CAR T cells.^29^, ^30^, ^31^ Our results are presented in Figure 2f. We did not observe a significant difference in transduction efficiencies (RD114: 37.1 ± 9.5%; VSV‐g: 34 ± 10.6%) or viability of the engineered primary T cells. Given the wide acceptance of VSV‐g pseudotyped lentivectors and our experience working with it, we continued using it in our process.

We also investigated the effect of cell concentration (Figure S3a), multiplicity of infection (MOI) (Figure S3b), transduction reaction volume (Figure S3c), and polybrene concentration (Figure S3d) on transducing primary T cells. Based on the tested range of each parameters studied and our prior experience,^21^ we developed a spinoculation process where 1 million primary T‐cells were transduced at an MOI of 10 in 500 μl with 8 μg/ml polybrene.

To further enhance the process yield, we investigated two different classes of chemical additives, antiviral inhibitors (AVIs) and latency reversal agents (LRAs). The results are detailed in Figure S4. The intracellular antiviral response impedes the transduction efficiency of primary T cells when lentivirus‐based vectors are used.^32^, ^33^ To address this issue, we tested the use of AVIs to suppress the intracellular immunity against infection from the lentiviral vectors and potentially increase the transduction yield. Inhibition of intracellular antiviral signaling has improved NK‐cell and T‐cell transduction.^32^, ^33^

We tested several AVIs for inhibiting three different antiviral pathways, and the results are shown in Figure S4a. These include (a) the TANK‐binding Kinase 1 (TBK1) pathway: BX795 and (5Z)‐7‐oxozeaenol; (b) the RNA‐dependent protein kinase (PKR) pathway: 2‐aminopurine (2‐AP) and C16; and (c) other pathways such as STAT (ruxolitinib) and Rho (Y‐27632) signaling. Concomitant treatment with AVIs for PKR or TBK1 pathways during T‐cell transduction increased transduction of primary T cells (Figure S4a). Although promising, the use of AVIs to generate cell therapy for clinical use requires further validation. For example, TBK‐1 inhibition has been associated with T‐cell dysfunction that compromises antitumor immunity.^34^

Figure S4b shows our results with LRAs as additives in the T‐cell transduction and expansion media. The LRAs facilitate unfolding of the chromatin structure that determines DNA accessibility^35^, ^36^ of the host genome and retroviral gene integration. A preferential bias for the site of gene integration is strongly displayed by gamma‐retroviruses, delta‐retroviruses, and lentiviruses with DNA insertion into transcriptionally active chromatin.^37^, ^38^ We tested a subset of LRAs such as protein kinase C agonists and/or its combination with inhibitors of bromo extra terminal or histone deacetylases for their ability to improve T‐cell transduction with large lentiviral constructs. The LRA romidepsin increased the percentage of the engineered T cells (55%) versus vehicle control (42%) (Figure S4b), but the percentage of live cells was only 40% compared to 75% in the control, rendering romidepsin unfit for use in combination with lentivectors. Although AVIs and LRAs may be useful for increasing yield, we are not integrating them in our process flow at this time.

We applied the knowledge gained from the above investigations to create an integrated process flow, and then validated it by transducing primary T cells from three human donors (i.e., *n* = 3). For improved transduction, we decided to use the approach of restricting the volume of transduction reaction. Indeed, we have previously used this approach of restricting the reaction volume to load nearly complete population of the 100 million primary T cells with nanoparticle‐based imaging agents within 10 min.^39^ We used three different methods to confine the volume of transduction reaction before adding IL‐2 to each reaction mix: (Method a) spinoculating in 0.5 ml at 800*g* in a well of a 24‐well plate for 1.5 h followed by incubating the reaction in a cell culture incubator (37°C, 5% CO_2_, 95% humidity) for another 14.5 h; (Method b) using a defined reaction volume of 1.0 ml in a well of a six‐well plate for 16 h in cell culture incubator; and (Method c) restricting the volume within a 0.1 ml drop in a well of a six‐well plate for 16 h in cell culture incubator. We transduced 1 × 10^6^ primary T cells with the lentivector particles at an MOI of 10 in serum‐free medium supplemented with 8 μg/ml polybrene and diluted the transduction reaction with complete growth medium supplemented with 50 U/ml IL‐2 after 16 h in all three methods. We assessed the process efficiency 5 days after the transduction (Figure 2g). The results showed 32% transduction at 44% viability with spinoculation (Method a); 53% transduction at 8% viability when using 1.0 ml reaction volume (Method b); and 60% transduction at 60% viability when restricting the reaction volume within the 0.1 ml drop (Method c). Limiting the reaction volume to increase lentivector‐to‐cell contact allowed Method c to produce ~2.5‐fold more engineered primary T cells compared to the more commonly used spinoculation method (Method a). Based on these results, we selected Method c, confining the transduction reaction within 0.1 ml drop, as part of the optimized production process.

### 2.2 Investigation of factors affecting expansion of functional engineered primary T‐cell‐based delivery system

**FIGURE 3:**
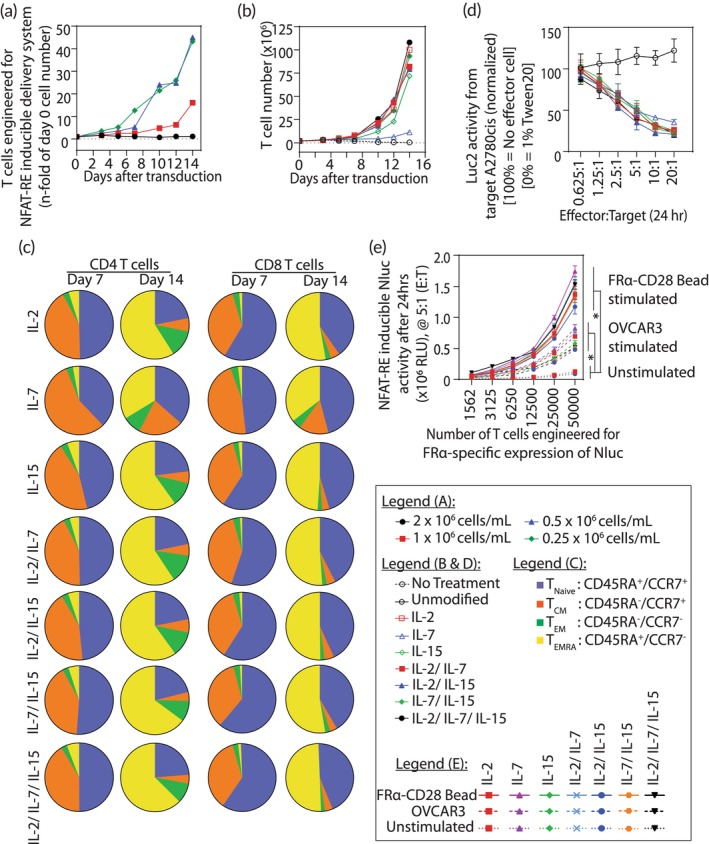
Factors affecting expansion of engineered primary T cells with delivery system. Numerical expansion of the engineered primary T cells was assessed (a) at different cell densities and (b) when supplemented with different cytokines (IL‐2, IL‐7, IL‐15, and combinations thereof). (c) Naïve/memory T cell phenotype (naïve [*T*_N_: CD45RA^+^/CCR7^+^], central memory [*T*_CM_: CD45RA^−^/CCR7^+^], effector memory [*T*_EM_: CD45RA^−^/CCR7^−^], and terminally differentiated effector memory [*T*_EMRA_: CD45RA^+^/CCR7^−^]) was assessed by flow cytometry in CD4 and CD8 T cells at day 7 and 14 of in vitro expansion using the same cytokine combinations. (d) FRα‐specific engineered primary T cells expanded in the same cytokine combinations induced cytolysis in FRα^+^Luc2‐2A‐E2Crimson^+^ A2780cis target cells in a dose‐dependent manner. (e) FRα‐specific engineered primary T cells expanded in the same cytokine combinations induced effector function, that is, the delivery function, as represented by the NFAT‐RE inducible NanoLuc (Nluc) reporter activity in a dose‐dependent manner. The FRα‐specific engineered primary T cells were stimulated at 5:1 effector‐to‐target ratio (E:T) by beads conjugated to the target FRα antigen and CD28 co‐stimulation molecules or by OVCAR3 cells that endogenously express FRα. All results are represented as mean ± SD. Statistical analysis and *p* values for (e) was determined by one‐way ANOVA and Tukey's multiple comparison test, *p < 0.05.

Figure 3 shows the effect of T‐cell density and cytokines for in vitro expansion of the primary T cells engineered with our cell‐based delivery system. Figure 3a shows the effect of cell density on numerically expanding these engineered primary T cells in the presence of 50 IU/ml IL‐2. In a 14‐day culture, the engineered T cells expanded ~45‐fold at cell densities of 0.5 × 10^6^ cells/ml and 0.25 × 10^6^ cells/ml, compared to the ~16‐fold expansion at 1 × 10^6^ cells/ml.

Figure 3b shows the expansion of engineered primary T cells in the presence of different cytokines (IL‐2, IL‐7, IL‐15, and combinations thereof). The engineered T cells were maintained at 0.5 × 10^6^ cells/ml by adding complete media every other day and including the respective cytokine supplements (50 IU/ml IL‐2, 25 IU/ml IL‐7, 25 IU/ml IL‐15). In a 14‐day culture, we observed a growth trend that ranged from ~70‐fold with IL‐15 to ~110‐fold when supplemented with combinations of IL‐2, IL‐7, and IL‐15.

Figure 3c shows phenotypic changes in the engineered primary T cells when cultured in different cytokine cocktails, as detailed in Figure 3b. We analyzed the CD4 and CD8 subsets at days 7 and 14 (see Figure S5 for CD4/CD8 ratios) for naïve (*T*_N_), central memory (*T*_CM_), effector memory (*T*_EM_), and terminally differentiated effector memory (*T*_EMRA_) phenotypes using the markers CD45RA and CCR7.^40^, ^41^, ^42^ At day 7, >90% of both CD4 and CD8 T cells were composed of *T*_N_ (CD45RA^+^/CCR7^+^ ~50%) and *T*_CM_ (CD45RA^−^/CCR7^+^ ~42%) compartments. After expansion from day 7 to day 14, as demonstrated in Figure 3b, the *T*_N_ compartment of CD4 T cells was reduced to less than 25%, and the *T*_CM_ compartment was reduced to ~6%. The expansion enriched the effector memory phenotypes characterized as *T*_EM_ (CD45RA^−^/CCR7^−^) from ~3% on day 7 to ~13% on day 14 and *T*_EMRA_ (CD45RA^+^/CCR7^−^) from ~5% on day 7 to ~60% on day 14.

We observed a similar trend in the CD8 T‐cell subset. On day 7, the CD8 T‐cell subset had a composition of ~58% *T*_N_, ~37% *T*_CM_, ~3% *T*_EM_, and ~2% *T*_EMRA_. On day 14, the composition was ~40% *T*_N_, ~4% *T*_CM_, ~3% *T*_EM_, and ~53% *T*_EMRA_. Except for the IL‐7‐supplemented culture, in which the *T*_N_ compartment of the engineered primary T cells had negligible change from day 7 to day 14, all other cytokines induced T‐cell expansion and had similar effects on the four phenotypes. This is in consensus with previous reports showing the use of IL‐7 for generating less differentiated CAR T cells that have stem‐like T‐phenotypes.^43^ The T cells showed notable enrichment of the *T*_EMRA_ compartment while the *T*_CM_ compartment was significantly reduced; this effect was least pronounced in the IL‐7‐supplemented culture. Although the IL‐7‐only culture showed reduced expansion at 11‐fold (Figure 3b), it produced a higher fraction of naïve‐ and central‐memory T cells known for superior antitumor effect. Indeed, IL‐7‐treated T cells with their high antigen‐stimulated proliferation potential along with persistence and superior effectiveness have been used to reduce the number of CAR T cells in a dose required to exert a clinical response.^44^ In addition to the proliferation, we observed that the cytolytic function of the primary T cells engineered into our delivery platform toward FRα‐antigen expressing A2780cis tumor cell line (Figure 3d) was proportional to the effector‐to‐target ratio (E:T) regardless of the cytokine composition used to expand different T‐cell cultures. The target specific cytolytic function of our FRα‐CAR T cells was further supported by two independent tumor cell lines (A2780cis and KPCY) engineered for FRα‐antigen expression compared to the respective antigen negative control (Figure S6a,b).

The engineered effector function, that is, the delivery function, as represented by the NFAT‐RE inducible NanoLuc (Nluc) reporter protein activity in the same T‐cell cultures, is shown in Figure 3e. The engineered primary T cells were expanded for 14 days and stimulated by beads (conjugated to the FRα antigen and anti‐CD28 antibody, i.e., FRα‐antigen/anti‐CD28) or by OVCAR3 cells (expressing endogenous FRα antigen). After 24 h of stimulation, we observed 2‐fold higher delivery function in these engineered primary T cells when FRα‐antigen/anti‐CD28 beads were used for stimulation compared to OVCAR3 cells, and a 6‐fold increase in the delivery function compared to that observed from the unstimulated engineered primary T cells.

Although the IL‐7‐expanded engineered primary T cells showed peak delivery function, it was not significantly different from the engineered primary T cells expanded with other cytokine combinations. Unlike the engineered primary T cells expanded in IL‐7 only, those expanded with the combination of IL‐7 and IL‐15 exhibited enhanced proliferation. Previous reports also support the use of IL‐7 and IL‐15 for the long‐term persistence and memory responses of the T cells^45^, ^46^, ^47^, ^48^; as such, IL‐7 and IL‐15 are currently being considered for use in cell therapy clinical trials.^49^ Therefore, we selected IL‐7 and IL‐15 as cytokine supplements for expanding our primary T cells engineered for cell‐based delivery.

To summarize, our development efforts to this point encompassed optimizing multiple process parameters and assessing their effects on the in vitro performance of our T‐cell‐based delivery platform. In particular, superior results were obtained when we activated the thawed T cells with anti‐CD3/CD28 Dynabeads (cell: bead = 1:3) for 24 h, transduced the activated T cells by increasing lentivector‐to‐T‐cell contact in 0.1 ml volume for 16 h, and expanded the transduced cells at 0.5 × 10^6^ cells/ml in *complete growth medium* supplemented with IL‐7 and IL‐15, with half‐media changes every 2–3 days for 14 days. These optimized parameters were employed in the subsequent in vivo validation studies below, and are described in detail in Section 4.

### 2.3 Functional validation of engineered primary T‐cell‐based delivery platform

**FIGURE 4:**
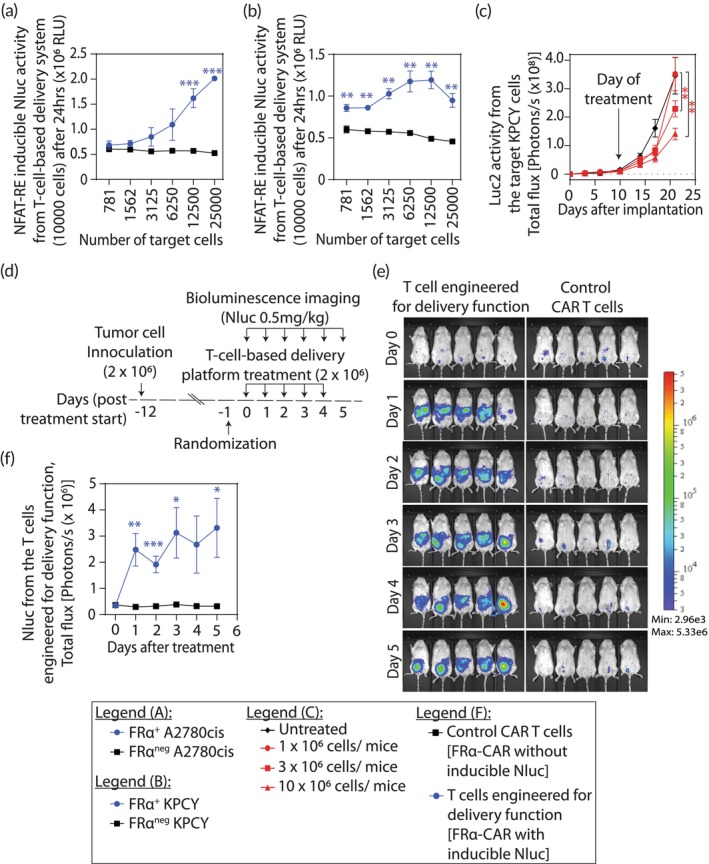
Functional validation of the primary T‐cell‐based delivery platform. (a, b) In vitro validation of target‐specific delivery function proportionate to the disease burden. FRα‐specific primary T cells engineered for the NFAT‐RE inducible delivery function showed proportionate increase in reporter activity when cocultured with target, (a) FRα^+^A2780cis and (b) FRα^+^KPCY cells, compared to their respective nontarget (FRα^neg^) control cells. (c) CAR T cells manufactured using the process developed for T‐cell‐based delivery platform reduced tumor burden. Tumor regression was observed in intraperitoneal (i.p.) KPCY tumors in NSG mice when treated with FRα‐specific CAR T cells in a dose‐dependent manner (*n* = 5 mice per group). Bioluminescence (Luc2 activity) from the i.p. tumors was used to assess the tumor burden in vivo. Statistical analysis was performed using two‐way ANOVA and Tukey's multiple comparison test. There was a statistically significant interaction between days and FRα‐specific CAR T cell doses on tumor burden (*F*(18, 96) = 4.595, *p* < 0.0001). (d–f) The primary T‐cell‐based delivery platform manufactured using the same process was functional in vivo in an antigen‐specific manner (*n* = 5 mice per group). FRα‐specific primary T cells engineered for the NFAT‐RE inducible delivery function were i.p. injected in i.p. FRα^+^A2780cis tumor‐bearing NSG mice at 24‐h interval for 5 days and NFAT‐RE inducible effector (Nluc) activity was measured for 6 days including the day of injection as a baseline to assess the delivery function. A control group was included to assess any background signal that may arise from using the Nluc substrate with Luc2^+^ tumor cells and injected with FRα‐specific primary CAR T cells (engineered without the NFAT‐RE inducible effector [Nluc]) to maintain equivalent tumor burden. (d) Schematic of dosing, treatment, and imaging schedules, (e) bioluminescent images, and (f) quantification. All results are represented as mean ± SEM. Statistical analysis and *p* values for (a), (b), and (f) were determined by multiple *t* test using Holm–Sidak method. *p < 0.05, **p < 0.01, and ***p < 0.001.

Figure 4 shows functional validation of our T‐cell‐based delivery platform. The target‐specific, delivery function proportionate to the disease burden was assessed in vitro by coculturing the FRα‐specific primary T cells engineered for the NFAT‐RE inducible delivery function against target cells, A2780cis (Figure 4a) and KPCY cells (Figure 4b). Compared to the nontarget cells, that is, antigen‐negative cells (FRα^neg^A2780cis, FRα^neg^KPCY), coculture with antigen‐positive target cells showed a proportionate and significant increase in delivery function, Nluc reporter activity, with increase in target cell number. However, the control cells (i.e., primary T cells engineered for the NFAT‐RE inducible delivery function but without CAR) did not exhibit the delivery function when cocultured with the same target and nontarget cells (Figure S6c).

Next, we ensured that the aforementioned procedure does not compromise the inherent cytolytic function of CAR T cells. KPCY2838c3 pancreatic ductal adenocarcinoma cells derived from KPCY mice were engineered to express human FRα antigen and Luc2 (FRα^+^Luc2^+^KPCY cells) for assessing tumor growth, and 0.5 × 10^6^ were intraperitoneally (i.p.) implanted in NSG mice. FRα‐CAR^+^ T cells (without the NFAT‐RE inducible Nluc reporter) were expanded for 16 days and injected i.p. to challenge 10‐day old FRα^+^Luc2^+^KPCY tumors. The results in Figure 4c show a dose‐escalation effect of the FRα‐CAR^+^T cells (1 × 10^6^, 3 × 10^6^, and 10 × 10^6^ FRα‐CAR^+^T cells) on tumor regression. Compared to the group with no treatment, we observed ~60% tumor regression on day 21 when treated with 10 × 10^6^ FRα‐CAR^+^T cells (*p* < 0.01) and ~35% by 3 × 10^6^ FRα‐CAR^+^T cells (*p* < 0.01).

We next manufactured FRα‐CAR^+^ T cells with the delivery function, that is, upon engaging the target FRα antigen, the FRα‐CAR activates the NFAT‐RE signaling pathway to induce the expression of desired protein. The experiment schedule is detailed in Figure 4d and the results are shown in Figure 4e,f. Then, 2 × 10^6^ FRα^+^Luc2^+^A2780cis cells were i.p. implanted in NSG mice. The 12‐day‐old xenograft tumors were i.p. treated with 2 × 10^6^ FRα‐CAR^+^ T cells (with NFAT‐RE inducible Nluc reporter) on days 0, 1, 2, 3, and 4. A control group was included to assess any background signal from using the Nluc substrate on Luc2^+^ tumor cells. This group was treated with i.p. injections of FRα‐CAR^+^ T cells without NFAT‐RE inducible Nluc reporter (control FRα‐CAR^+^T cells) to maintain an equivalent tumor burden. The effector (Nluc) activity (Figure 4e) was measured and quantified (Figure 4f) at baseline (day 0) as well as on days 1, 2, 3, 4, and 5. A significant increase in engineered effector activity (i.e., delivery function) was observed in the group treated with the FRα‐CAR^+^ T cells with the delivery function (i.e., with NFAT‐RE inducible Nluc reporter), confirming the target‐inducible in situ delivery function in the engineered primary T cells.

## 3 DISCUSSION

In this work, we used primary T cells to develop a cell‐based platform that can be used for site‐specific delivery of protein‐based drugs. Our platform delivery system utilizes the T‐cell's activation machinery for in situ synthesis, so that the cell‐mediated synthesis of desired proteins is proportionate to the disease burden. The site‐specific and proportionate synthesis of desired biologics offers the potential to overcome morbidity issues that can arise from excess systemic infusion of such drugs, and prevents the development of resistance to these drugs when used in lower amounts. We also conducted a pilot exploration of AVIs and LRAs to improve the lentivector transduction yield and increase the starting cell number so as to shorten dose‐manufacturing time, thereby improving the affordability of T‐cell therapies. The use of these additives, AVIs and LRAs, may also reduce cell exhaustion by decondensing the chromatin structure.

Although first‐order drug‐delivery systems (e.g., liposomes, nanocarries, dendrimers, hydrogels, microparticles) offer a controlled release of drugs, their application is still limited by their short half‐life in vivo, requirement for multiple infusions, and potential toxicity due to their systemic presence.^50^, ^51^, ^52^ Our T‐cell‐based delivery platform represents a substantive departure from this status quo. This is because T cells chemotactically extravasate through multiple solid tissues to the disease sites and engage with the target cells through the antigen‐specific CAR.^53^, ^54^ At the single‐cell level, this cues the activation pathway in a binary (on/off) event independent of the antigen density on the surface of the disease cell,^9^, ^10^, ^11^, ^12^ and executes a parallel program resulting in clonal expansion of the activated T cells.^55^, ^56^ The integrated effect is a clonal CAR T cell population proportionate to the number of target cells. Our T‐cell‐based drug delivery system is engineered to leverage this biology of the T cell. T cells migrate to disease sites with cellular resolution and, upon recognizing the target cells with molecular specificity, can synthesize protein‐based biologics proportionate to the disease burden. It is therefore a living cell‐based in vivo vector engineered into a stable zero‐order drug delivery system.^52^ Unlike the first‐order drug delivery systems, it can enable sustained in situ production of complex biologic drugs for executing a broad range of effector functions.

Additionally, the first‐order drug‐delivery approaches are primarily based on synthetic materials and are thus rapidly cleared by the mononuclear phagocyte system.^57^ In contrast, our cell‐based system utilizes T cells that have been found to persist in vivo for more than a decade.^58^, ^59^ In fact, recent findings in mice concluded that the primary T cell, when passaged in vivo in new mice, can last four times longer than the lifespan of the host species and expand at least 10^40^‐fold.^60^ This obviates the need for re‐dosing even in case of disease relapse. Therefore, while the potential to target the basal expression of the target antigen exists, as seen in the case of CAR T cells, our primary T cell‐based delivery platform presents a pioneering and universal technology. It facilitates the delivery of intricate biologics over extended periods without the need for multiple infusions. As a result, this platform technology opens new horizons for treating a variety of diseases. For example, we have already employed immortalized cell lines to establish the feasibility of using the synthetic pathway for localized delivery of various type‐I and type‐III interferons for targeting SARS‐CoV‐2.^22^, ^24^ Our current efforts are geared toward applying our antigen‐specific primary T‐cell‐based system for in situ synthesis of such biologic drugs to exert their therapeutic effects within the local tumor microenvironment. Our parallel efforts, in continuation of this work, also include improving our delivery system so as to increase the amount of protein‐based drugs produced from a single cell while reducing its expression that may occur under unstimulated conditions.

## 4 METHODS

### 4.1 Materials and reagents

The key resources table lists sources for all materials, supplies, services, and equipment used in our investigative processes.

Key Resources Table

**Table:** 

REAGENT OR RESOURCE	SOURCE	IDENTIFIER (CAT#)
*Biological samples*		
Human primary CD3 T cells	Stanford Blood Center	A1020
A2780cis	Sigma‐Aldrich	93112517
OVCAR3	ATCC	HTB‐161
KPCY2838c3	Kerafast	EUP013‐FP
HEK293T/17	ATCC	CRL‐11268
NOD.Cg‐Prkdc^scid^ Il2rg^tm1Wjl^/SzJ (NSG)	Jackson Laboratory	005557
*Chemicals*, *peptides*, *recombinant proteins*		
Fetal bovine serum (FBS)	Sigma‐Aldrich	F2442‐500ML
Dimethyl sulfoxide (DMSO)	Sigma‐Aldrich	D2650‐100mL
GlutaMAX	Gibco	35050‐061
Penicillin streptomycin solution	Corning	30‐002‐Cl
Human IL‐2	Miltenyi Biotec	130‐097‐748
Human IL‐15	PeproTech	200‐15‐50UG
Human IL‐7	PeproTech	200‐07‐50UG
Dynabead biotin binder	Invitrogen	11047
Phorbol 12‐myristate 13‐acetate (PMA)	Sigma‐Aldrich	P1585
Ionomycin (Io)	Sigma‐Aldrich	I0634
DMEM media	Corning	10‐013‐CV
Transporter 5	Polysciences, Inc.	26008‐5
RPMI1640	Corning	10‐040‐CV
Phosphate buffered saline (PBS) without Ca^+2^ and Mg^+2^	Corning	21‐040‐CV
Puromycin dihydrochloride	ThermoFisher Scientific	A1113803
2‐Aminopurine (2‐AP)	Combi‐Blocks	OR‐1074
C16	EMD Millipore	527451‐5MG
BX‐795	Cayman Chemical	14932
(5z)‐7‐oxozeaenol	Cayman Chemical	17459
Y‐27632	Tocris	1254
Ruxolitinib	Cayman Chemical	11609
Romidepsin	Cayman Chemical	17130
Bryostatin	EMD Millipore	203811‐10UG
Prostratin	Cayman Chemical	10272
(+)‐JQ1	Cayman Chemical	11187
Biotinylated human folate receptor 1 (FOLR1‐His Tag‐Avi Tag)	Acro Biosystems	FO1‐H82E2
d‐Luciferin	Perkin Elmer	122799
Acridine orange propidium iodide (AOPI) staining solution	Nexcelom	CS2‐0106‐5 ml
Cell‐staining buffer	Biolegend	420201
RetroNectin	Takara Bio	T100B
*Antibodies*		
Biotin antihuman CD3	Biolegend	317320
Biotin antihuman CD28	Biolegend	302904
PerCR/Cy5.5 antihuman CD3 antibody	Biolegend	300328
LIVE/DEAD Fixable Aqua Dead Cell Stain Kit	Life Technologies	L34966
APC‐streptavidin	Biolegend	405207
Brilliant violet 510 antihuman CD25	Biolegend	302639
Alexa Fluor 700 antihuman CD69	Biolegend	310321
Human naïve/memory T‐cell ID panel kit	Biolegend	362201
*Plasmids*		
Lentivector transfer plasmid	System Biosciences	CD510B‐1
psPAX2	Addgene	12260
pMD2.G	Addgene	12259
pAdVAntage	Promega	E1711
*Critical commercial assays*		
Nano‐Glo assay	Promega	N1120
One‐Glo assay	Promega	E6110
*Other materials*		
T150 flasks	Corning	430825
Hera 150i CO_2_ incubator	Thermo Scientific	50116047
Cellaca slides	Nexcelom	CHT4‐PD100‐003
Six‐well incubation plate	Fisher scientific	FB012927
96‐well plate	Greiner bio‐one	655083
0.45 μm filter	Corning	430514
500 ml collection bottle	Corning	430282
Konical ultracentrifugation tube	Beckman Coulter	C14291
Nexcelom K2 cellometer	Nexcelom	
DynaMag‐2 sample rack	Life Technologies	12322D
BD FACS Symphony A3 cell analyzer	BD Biosciences	
EnVision Multilabel Plate Reader	Perkin Elmer	Model 2104‐0010A
AMI HTX Spectral instrument	Spectral Instruments Imaging	
IVIS Lumina X5 imaging system	Perkin Elmer	CLS148590
Optima XPN‐90 ultracentrifuge	Beckman Coulter	
*Software*		
FlowJo software	FlowJo LLC	
Aura Image software	Spectral Instruments Imaging	
Living Image software	Perkin Elmer	128110
SnapGene software	GSL Biotech LLC	
Prism version 8.0	GraphPad Software	

### 4.2 Preparations

Transfer plasmids with different genetic payloads were designed in SnapGene software and subcloned into the lentivector plasmid. Epoch Life Science, Inc. (Missouri City, TX) provided plasmid preparation services (chemical synthesis of DNA insert sequences, subcloning into respective vector backbones, and the amplification). Target cells (FRa^+^A2780cis [sex: female], FRa^+^OVCAR3 [sex: female]) engineered to express modified firefly luciferase (Luc2), as detailed in our previous reports,^13^, ^61^ were maintained in *RPMI media* (RPMI1640, 10% heat‐inactivated FBS, and 1X penicillin streptomycin solution). The mouse pancreatic tumor cell line (KPCY2838c3 [sex: female]) was maintained in *DMEM media* (DMEM, 10% heat‐inactivated FBS, 1x GlutaMAX, and 1X penicillin streptomycin solution). Phosphate buffered saline (PBS) without Ca^+2^ and Mg^+2^ was used to minimize cell clumping. When applicable, puromycin *N*‐acetyltransferase was used as a selection marker and puromycin dihydrochloride (Puromycin) was used for selecting stable cell lines. A chemical activation of T‐cells was achieved by treatment with 1 μM phorbol 12‐myristate 13‐acetate and 30 nM ionomycin (PMA/Io). Biotinylated human folate receptor 1 (FRα) protein was used to analyze FRα CAR expression on engineered primary T cells.

### 4.3 Method for producing lentivector particles

Lentivector particles were produced as previously reported.^21^ Lentivirus manufacturing and its use in engineering cells were performed at SRI International following the guidelines of the approved Biological Use Authorization (BUA 17‐05). Briefly, lentivector particles were prepared by packaging the corresponding transfer plasmid using second‐generation lentivector system. HEK293T/17 (sex: female) producer cells (12 × 10^6^) were seeded into tissue culture treated T150 flasks in 21 ml complete DMEM supplemented with 10% heat‐inactivated FBS and 1X penicillin streptomycin solution and placed in a cell culture incubator (37°C, 5% CO_2_, 95% humidity). After 24 h, transfer plasmid was co‐transfected with second generation packaging plasmids (psPAX2, pMD2.G), and pAdVAntage plasmid at 4:3:1:0.4 wt‐ratio, respectively (transfer plasmid: 12 μg, pxPAX2: 9 μg, pMD2.G: 3 μg, pAdV: 1.2 μg). Transporter 5 transfection reagent was used following the manufacturer's protocol (100 μl). Cell culture supernatant enriched with pseudo‐viral particle was collected and replenished every 24 h for 3 days (30 ml). The lentivector‐enriched cell culture supernatant was clarified using a 0.45 μm filter. The supernatant was clarified by transferring it to a polypropylene Konical ultracentrifugation tube and centrifuging at 20,700*g* in an SW32‐Ti rotor using a Beckman Coulter Optima XPN‐90 ultracentrifuge at 4°C for 2 h. The resulting pellet was resuspended in 400 μl serum‐free RPMI and aliquoted. Based on our experience, we expect to achieve an MOI of 10 when the lentivector particles produced in this process are used to transduce 1 × 10^6^ cells. The lentivector aliquots were stored at −80°C until use.

### 4.4 Method for engineering primary T cells

The primary T‐cells engineered with NFAT‐RE inducible drug delivery system and used in the in vivo validation studies (Figure 4) were manufactured using the procedures resulting from the cumulative developments reported in this manuscript. Human primary CD3 T cells were purchased from the Stanford Blood Center (Palo Alto, CA). The T cells were immediately counted and used fresh or were cryostocked using *freezing media* (90% heat‐inactivated fetal bovine serum [FBS] and 10% dimethyl sulfoxide) in liquid nitrogen for future use. Biotin anti‐human CD3 and Biotin anti‐human CD28 antibody were loaded on Dynabead Biotin Binder paramagnetic particles following the manufacturer's instructions (anti‐CD3/CD28 Dynabeads). Human primary T cells were thawed (Day 0), resuspended in *complete growth medium*, and activated by anti‐CD3/CD28 Dynabeads (cell: bead = 1:3). After 24 h (Day 1), 1 × 10^6^ activated primary T cells were transduced with the appropriate lentivector particles resuspended in 0.1 ml volume of serum‐free RPMI at an MOI of ~10 and in the presence of 8 μg/ml polybrene. The 0.1 ml aliquots of transduction reaction mix were placed as drops in a tissue culture treated six‐well plate and placed in a cell culture incubator (37°C, 5% CO_2_, 95% humidity) for 16 h. After 16 h of incubation (~Day 2), the transduced primary T cells were cultured at 1 × 10^6^ cells/ml in *complete growth medium* supplemented with recombinant human IL‐7 (25 IU/ml), recombinant human IL‐15 (25 IU/ml), and 8 μg/ml polybrene. The cells were counted after another 24 h (Day 3) and every other day thereafter using acridine orange and propidium iodide staining in a Nexcelom K2 cellometer. They were maintained at a concentration of 0.5 × 10^6^ cells/ml in *complete growth medium* supplemented with recombinant human IL‐7 (25 IU/ml) and recombinant human IL‐15 (25 IU/ml) and the media was replaced every 2–3 days with half media changes. No polybrene was added on Day 3 and beyond.

### 4.5 Approach for improving the production of primary T‐cell‐based delivery system

We deviated from the above process when exploring the factors that could improve the production of our cell‐based delivery system. For transduction optimization, we varied the duration of activation (Figure 2a–c), cell‐to‐bead ratio (Figure 2a–d), construct size (Figure 2e), pseudotyped lentivectors (Figure 2f), and transduction methods (Method a, Method b, Method c) (Figure 2g). For transduction optimization, we also varied the (1) concentration of activated primary T cells (cell count was varied in volume of 0.5 ml) (Figure S3a), (2) MOI (cell count = 1 × 10^6^, volume = 0.5 ml) (Figure S3b), transduction reaction volumes (cell count = 1 × 10^6^, MOI = 10) (Figure S3c), and polybrene concentrations (cell count = 1 × 10^6^, MOI = 10, volume = 0.5 ml) (Figure S3d). To further explore the effect of AVIs (PKR inhibitor 2‐aminopurine [2‐AP] and C16, TANK‐binding kinase inhibitors BX‐795 and [5z]‐7‐oxozeaenol, rock inhibitor Y‐27632 and STAT inhibitor ruxolitinib) (Figure S4a) and LRAs (Romidepsin, Bryostatin, Prostratin and [+]‐JQ1) (Figure S4b), we varied the transduction buffer composition by adding these chemicals. For optimizing the expansion of transduced T cells, we varied starting cell concentration of the transduced cells (Figure 3a), and growth cytokines (Figure 3b–e).

### 4.6 Flow cytometry analysis

The production yield of engineered primary T cells was determined by assessing the expression of FRα‐CAR on the T cells engineered for drug delivery (% FRα‐CAR^+^ T cells) and cell viability. Five days after transduction, ~1 × 10^6^ T cells were collected and debeaded by keeping the T‐cell suspension on a DynaMag‐2 sample rack for 2 min to remove the Dynabead biotin binder particles. The debeaded T cells were washed in cell‐staining buffer and stained for 1 h at 4°C using an antibody cocktail containing biotinylated human FRα protein (FOLR1‐His Tag‐Avi Tag), PerCR/Cy5.5 antihuman CD3 antibody and the LIVE/DEAD Fixable Aqua Dead Cell Stain Kit. The cells were washed, and a secondary staining was performed for 1 h at 4°C using APC‐streptavidin. The samples were washed, resuspended in 200 μl Cell Staining Buffer, and analyzed with a BD FACS Symphony A3 (BD Biosciences). The data were further processed using FlowJo software. To determine T‐cell activation (Figure 2a–c), a single‐staining step protocol was followed whereby the debeaded T cells were washed and stained for 1 h at 4°C using an antibody cocktail containing PerCR/Cy5.5 antihuman CD3 antibody, brilliant violet 510 antihuman CD25 antibody, and Alexa Fluor 700 antihuman CD69 antibody. Gating was performed using unstained control sample upon fluorescence compensation. To assess the naïve/memory phenotype of expanded T cells (Figure 3f), we utilized the human naïve/memory T‐cell ID panel kit. All samples were analyzed using BD FACS Symphony A3 (BD Biosciences) and the data were further processed using FlowJo software.

### 4.7 In vitro assessment of cytolytic function of the engineered T cells (engineered for delivery function)

FRα‐CAR^+^ T‐cell (with NFAT‐RE inducible delivery function) were cocultured with target (FRα^+^Luc2‐2A‐E2Crimson^+^ A2780cis) cells (2500 cells) in 200 μl of *complete growth medium* in a single well of a 96‐well plate. After a 24‐h coculture, the manufacturer's protocol was followed to measure the reporter activity, that is, Luc2 activity in the A2780cis cells using One‐Glo assay. Briefly, the Luc2 substrate was diluted in the cell lysis buffer provided with the One‐Glo assay and added to coculture in the 96‐well plate for assessing Luc2 activity. Following a brief incubation period of 10 min, the bioluminescence was read on a microplate reader.

### 4.8 In vitro assessment of delivery function of the engineered T cells (engineered for delivery function)

FRα‐CAR^+^ T‐cell (with NFAT‐RE inducible delivery function) were cocultured with the targets (OVCAR3 or FRα‐antigen/anti‐CD28 Dynabeads) at an effector‐to‐target ratio (E:T) of 5:1, in 200 μl of *complete growth medium* in a single well of a 96‐well plate. After a 24‐h coculture, the manufacturer's protocol was followed to measure the reporter activity, that is, NanoLuc (Nluc) activity in the engineered primary T cells using Nano‐Glo assay. Briefly, the Nluc substrate was diluted in the cell lysis buffer provided with the Nano‐Glo assay and added to the coculture in 96‐well plates to assess the enzyme (Nluc) activity. Following a brief incubation period of 3 min, the bioluminescence was read on a microplate reader.

### 4.9 Challenge of in vivo tumor with CAR T cells (used as control cells, i.e., engineered without the NFAT‐RE inducible delivery system)

The in vivo tumor challenge mouse study (Figure 4c) was performed at the Molecular Medicine Research Institute in accordance with the guidelines from the Institutional Animal Care and Use Committee (Approval # 22‐001). Twenty 6–8‐week‐old male NOD.Cg‐Prkdc^scid^ Il2rg^tm1Wjl^/SzJ (NSG) mice were purchased from The Jackson Laboratory. The KPCY2838c3 cells, engineered to express human FRα, Luc2, and E2Crimson using lentiviral vector (FRα^+^Luc2‐2A‐E2Crimson^+^ KPCY2838c3 cells), were selectively expanded in the presence of puromycin. The NSG mice were anesthetized and 1 × 10^5^ FRα^+^Luc2‐2A‐E2Crimson^+^ KPCY2838c3 cells in 100 μl 1x PBS were i.p. implanted. After 10 days, the mice were randomized into four groups (*n* = 5 each) and three groups were treated with doses of 1 × 10^6^, 3 × 10^6^, or 10 × 10^6^ FRα‐CAR^+^ T cells. The fourth untreated group was used for negative control. The tumor growth was monitored every 3–4 days using i.p. injected 150 mg d‐luciferin per kg of mouse dissolved in 1x PBS. The luminescence imaging was performed in an AMI HTX Spectral instrument with a 60‐s exposure. The data were quantified by analysis of the region‐of‐interest (ROI) using Aura Image software. The tumor luminescence is plotted as the mean ± SEM of total flux (photons/s) against days after tumor implantation.

### 4.10 In vivo validation of delivery function of the engineered T cells (experimental cells, i.e., engineered for delivery function with NFAT‐RE delivery system)

The in vivo validation of our T‐cell based delivery system was performed in mice at SRI International in accordance with the guidelines from the Institutional Animal Care and Use Committee (Approval # 22001). Then, 6–8‐week‐old female NOD.Cg‐Prkdc^scid^ Il2rg^tm1Wjl^/SzJ (NSG) mice were purchased from The Jackson Laboratory. After mandatory quarantine, the NSG mice were anesthetized and 2 × 10^6^ FRα^+^Luc2‐2A‐E2Crimson^+^ A2780cis cells in 100 μl 1x PBS were i.p. implanted. The tumor growth was monitored every 3–4 days for the next 12 days using i.p. injected 150 mg d‐Luciferin per kg of mouse dissolved in 1x PBS. At 11 days after implantation, the mice were randomized into two groups (*n* = 5 each). The two groups were then treated with 2 × 10^6^ primary T cells engineered for delivery function (i.e., FRα‐CAR with NFAT‐RE inducible Nluc reporter) or the control T cells (FRα‐CAR only, i.e., without NFAT‐RE inducible Nluc reporter) every day for 5 days. The bioluminescent reporter (Nluc) activity was determined by i.p. injection of the Nano‐Glo substrate (1:20 dilution of the substrate in 1x PBS, equivalent to 0.5 mg per kg of mouse) on days 0, 1, 2, 3, 4, and 5 after treatment. Imaging was performed in an IVIS Lumina X5 imaging system. The data were quantified by analysis of the ROI using Living Image software. The tumor luminescence is plotted as the mean ± SEM of total flux (photons/s) against days after treatment.

### 4.11 Statistical analysis

Results are expressed as an arithmetic mean ± SD if not otherwise stated. For each run, each sample was measured in a technical replicate. Values of *p* < 0.05 were considered to indicate statistical significance (represented as *p < 0.05, **p < 0.01, ***p < 0.001, and ****p < 0.0001), as determined using ANOVA with Tukey's multiple comparison test, or as specified. Analyses were performed using Prism version 8.0 (GraphPad Software).

## AUTHOR CONTRIBUTIONS

Parijat Bhatnagar was the principal investigator, designed the experimental investigations, analyzed the results, and wrote the manuscript. Harikrishnan Radhakrishnan designed the experimental investigations, performed the experiments, analyzed the results, and wrote the manuscript. Harold S. Javitz was the co‐investigator, designed the statistical tests, and analyzed the results. Sherri L. Newmyer and Marvin A. Ssemadaali conducted experiments. All authors read and contributed to preparation of the manuscript.

## FUNDING INFORMATION

Research reported in this publication was supported in part by the National Institute of Biomedical Imaging and Bioengineering (DP2EB024245: NIH Director's New Innovator Award Program, Office of the NIH Director, https://commonfund.nih.gov/newinnovator); the National Cancer Institute (R21CA236640 and R33CA247739) of the National Institutes of Health (NIH); and the Defense Advanced Research Projects Agency (DARPA) (D19AP00024: DARPA Young Faculty Award, https://www.darpa.mil/work-with-us/for-universities/young-faculty-award).

## CONFLICT OF INTEREST STATEMENT

The authors declare no competing financial interests.

## Supporting information


**FIGURE S1.** Strategy for evaluating CD3 T cell activation. (a) Schematic of the gating strategy used for assessing early (CD69^+^CD25^−^), peak (CD69^+^CD25^−^), and late (CD69^−^CD25^+^) activated CD3 T cells by flow cytometry. (b) Representative plots showing CD69 and CD25 expression in stimulated vs. non‐stimulated CD3 T cells.
**FIGURE S2**. Schematic of the genetic payloads. (a) chimeric antigen receptor (CAR) only, 5.6 kb and (b) T‐cell‐based delivery system comprising of CAR and NFAT‐RE inducible transgene, 7.2 kb.
**FIGURE S3**. Additional factors affecting transduction of primary T cells with lentivectors. FRα‐CAR expression (% FRα‐CAR^+^ T cells on left y‐axis) and T‐cell viability (% Viability on right y‐axis) were assessed by flow cytometry after varying factors affecting transduction: (a) T‐cell concentration in transduction reaction, (b) lentivector multiplicity of infection (MOI), (c) transduction reaction volume, and (d) polybrene concentration. Transduction efficiency was determined after 5 days. All results are represented as mean ± SD.
**FIGURE S4**. Exploratory screen of chemical additives for improving transduction of primary T cells with lentivectors. FRα‐CAR expression (% FRα‐CAR^+^ T cells on left y‐axis) and T‐cell viability (% Viability on right y‐axis) was assessed by flow cytometry after concomitant treatment with (a) antiviral inhibitors (AVIs), and (b) latency reversal agents (LRAs) during lentivector transduction. Transduction efficiency was determined after 5 days. All results are represented as mean ± SD.
**FIGURE S5**. Change in the proportion of CD3 T cell subsets in response to cytokines. The CD4/CD8 ratio was assessed by flow cytometry in CD3 T cells at day 7 and 14 of in vitro expansion when growth media was supplemented with different cytokines (IL‐2, IL‐7, IL‐15, and combinations thereof ).
**FIGURE S6**. Antigen‐specific cytolysis and NFAT‐RE inducible delivery function. FRα‐specific CAR T cells manufactured using the new process induced cytolysis in (a) FRα^+^Luc2‐2A‐E2Crimson^+^ KPCY and (b) FRα^+^Luc2‐2A‐E2Crimson^+^ A2780cis target cells in a dose‐dependent manner, compared to their respective antigen negative cells. (c) Nluc activity from primary T cells engineered without CAR but with NFAT‐RE inducible delivery function, when cocultured with antigen‐positive target cells for 24 h, did not exhibit cytolytic activity compared to antigen negative cells.

**Data S1:** Supporting information.

## Data Availability

All data used to draw conclusions from our work are present in the article and supporting information.
