# Correction to “Primary T‐cell‐based delivery platform for in vivo synthesis of engineered proteins”

**DOI:** 10.1002/btm2.10658

**Published:** 2024-03-10

Radhakrishnan H, Newmyer SL, Ssemadaali MA, Javitz HS, Bhatnagar P. Primary T‐cell‐based delivery platform for in vivo synthesis of engineered proteins. Bioeng Transl Med. 2024; 9(1):e10605. doi:10.1002/btm2.10605

Unlike the remainder of the manuscript where the data have been developed using pan CD3 T cells, Figure 4d–f used CD4 T cells. We apologize for this error. Corrections have been disclosed in the following places:

1. **Figure 4d–f labels, edited to indicate the use of CD4 T cells**

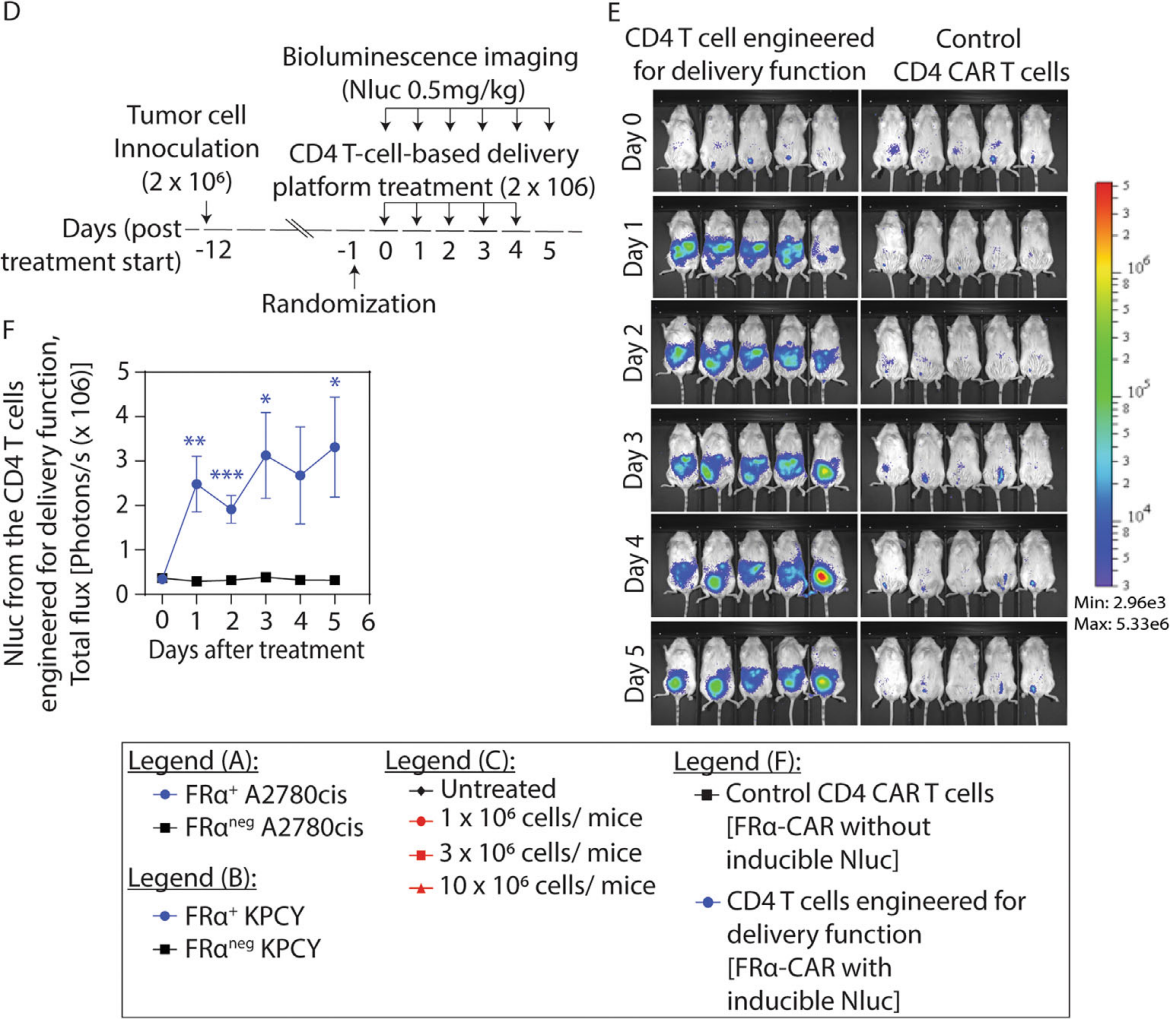

2 **Figure 4 caption, edited to indicate the use of CD4 T cells**

**FIGURE 4.** Functional validation of the primary T‐cell‐based delivery platform. (a, b) In vitro validation of target‐specific delivery function proportionate to the disease burden. FRα‐specific primary T cells engineered for the NFAT‐RE inducible delivery function showed proportionate increase in reporter activity when cocultured with target, (a) FRα^+^A2780cis and (b) FRα^+^KPCY cells, compared to their respective nontarget (FRα^neg^) control cells. (c) CAR T cells manufactured using the process developed for T‐cell‐based delivery platform reduced tumor burden. Tumor regression was observed in intraperitoneal (i.p.) KPCY tumors in NSG mice when treated with FRα‐specific CAR T cells in a dose‐dependent manner (*n* = 5 mice per group). Bioluminescence (Luc2 activity) from the i.p. tumors was used to assess the tumor burden in vivo. Statistical analysis was performed using two‐way ANOVA and Tukey's multiple comparison test. There was a statistically significant interaction between days and FRα‐specific CAR T‐cell doses on tumor burden (*F*[18, 96] = 4.595, *p* < 0.0001). (d–f) The primary T‐cell‐based delivery platform manufactured using the same process was functional in vivo in an antigen‐specific manner (*n* = 5 mice per group). FRα‐specific primary CD4 T cells engineered for the NFAT‐RE inducible delivery function were i.p. injected in i.p. FRα^+^A2780cis tumor‐bearing NSG mice at 24‐h interval for 5 days and NFAT‐RE inducible effector (Nluc) activity was measured for 6 days including the day of injection as a baseline to assess the delivery function. A control group was included to assess any background signal that may arise from using the Nluc substrate with Luc2^+^ tumor cells and injected with FRα‐specific primary CD4 CAR T cells (engineered without the NFAT‐RE inducible effector [Nluc]) to maintain equivalent tumor burden. (d) Schematic of dosing, treatment, and imaging schedules, (e) bioluminescent images, and (f) quantification. All results are represented as mean ± SEM. Statistical analysis and *p* values for (a), (b), and (f) were determined by multiple *t* test using Holm–Sidak method. *p < 0.05, **p < 0.01, and ***p < 0.001.

3 **A result in Section 2.3, edited to indicate the use of CD4 T cells**

Using primary CD4 T cells, we next manufactured FRα‐CAR^+^ T cells with the delivery function, that is, upon engaging the target FRα antigen, the FRα‐CAR activates the NFAT‐RE signaling pathway to induce the expression of desired protein. The experiment schedule is detailed in Figure 4d and the results are shown in Figure 4e,f. Then, 2 × 10^6^ FRα^+^Luc2^+^A2780cis cells were i.p. implanted in NSG mice. The 12‐day‐old xenograft tumors were i.p. treated with 2 × 10^6^ FRα‐CAR^+^ primary CD4 T cells (with NFAT‐RE inducible Nluc reporter) on Days 0, 1, 2, 3, and 4. A control group was included to assess any background signal from using the Nluc substrate on Luc2^+^ tumor cells. This group was treated with i.p. injections of FRα‐CAR^+^ primary CD4 T cells without NFAT‐RE inducible Nluc reporter (control FRα‐CAR^+^T cells) to maintain an equivalent tumor burden. The effector (Nluc) activity (Figure 4e) was measured and quantified (Figure 4f) at baseline (Day 0) as well as on days 1, 2, 3, 4, and 5. A significant increase in engineered effector activity (i.e., delivery function) was observed in the group treated with the FRα‐CAR^+^ T cells with the delivery function (i.e., with NFAT‐RE inducible Nluc reporter), confirming the target‐inducible in situ delivery function in the engineered primary T cells.

4 **Addition to the Key Resource Table in Section 4.1, to indicate the source of CD4 T cells**

**Table:** 

Human primary CD4 T cells	Stanford Blood Center	A1015

5 **A method in Section 4.10, edited to indicate the use of CD4 T cells**

**4.10 In vivo validation of delivery function of the engineered T cells (engineered for delivery function with NFAT‐RE delivery system).** The in vivo validation of our T‐cell based delivery system was performed in mice at SRI International in accordance with the guidelines from the Institutional Animal Care and Use Committee (Approval # 22001). Six‐ to 8‐week‐old female NOD.Cg‐Prkdc^scid^ Il2rg^tm1Wjl^/SzJ (NSG) mice were purchased from The Jackson Laboratory. After mandatory quarantine, the NSG mice were anesthetized and 2 × 10^6^ FRα^+^Luc2‐2A‐E2Crimson^+^A2780cis cells in 100 μL 1× PBS were i.p. implanted. The tumor growth was monitored every 3–4 days for the next 12 days using i.p. injected 150 mg d‐Luciferin per kg of mouse dissolved in 1× PBS. At 11 days after implantation, the mice were randomized into two groups (*n* = 5 each). The two groups were then treated with 2 × 10^6^ primary CD4 T cells engineered for delivery function (i.e., FRα‐CAR with NFAT‐RE inducible Nluc reporter) or the control primary CD4 T cells (FRα‐CAR only, i.e., without NFAT‐RE inducible Nluc reporter) every day for 5 days. The bioluminescent reporter (Nluc) activity was determined by i.p. injection of the Nano‐Glo® substrate (1:20 dilution of the substrate in 1× PBS, equivalent to 0.5 mg per kg of mouse) on Days 0, 1, 2, 3, 4, and 5 after treatment. Imaging was performed in a IVIS Lumina X5 imaging system. The data were quantified by analysis of the ROI using Living Image software. The tumor luminescence is plotted as the mean ± SEM of total flux (photons/s) against days after treatment.

