# Supplementary material for: Primary T‐cell‐based delivery platform for in vivo synthesis of engineered proteins
Source: Bioeng Transl Med. 2023 Oct 7;9(1):e10605. doi: 10.1002/btm2.10605 (PMC10771566; doi:10.1002/btm2.10605)
Supplement: Supplementary file 1 — FIGURE S1. Strategy for evaluating CD3 T cell activation. (a) Schematic of the gating strategy used for assessing early (CD69+CD25−), peak (CD69+CD25−), and late (CD69−CD25+) activated CD3 T cells by flow cytometry. (b) Representative plots showing CD69 and CD25 expression in stimulated vs. non‐stimulated CD3 T cells. FIGURE S2. Schematic of the genetic payloads. (a) chimeric antigen receptor (CAR) only, 5.6 kb and (b) T‐cell‐based delivery system comprising of CAR and NFAT‐RE inducible transgene, 7.2 kb. FIGURE S3. Additional factors affecting transduction of primary T cells with lentivectors. FRα‐CAR expression (% FRα‐CAR+ T cells on left y‐axis) and T‐cell viability (% Viability on right y‐axis) were assessed by flow cytometry after varying factors affecting transduction: (a) T‐cell concentration in transduction reaction, (b) lentivector multiplicity of infection (MOI), (c) transduction reaction volume, and (d) polybrene concentration. Transduction efficiency was determined after 5 days. All results are represented as mean ± SD. FIGURE S4. Exploratory screen of chemical additives for improving transduction of primary T cells with lentivectors. FRα‐CAR expression (% FRα‐CAR+ T cells on left y‐axis) and T‐cell viability (% Viability on right y‐axis) was assessed by flow cytometry after concomitant treatment with (a) antiviral inhibitors (AVIs), and (b) latency reversal agents (LRAs) during lentivector transduction. Transduction efficiency was determined after 5 days. All results are represented as mean ± SD. FIGURE S5. Change in the proportion of CD3 T cell subsets in response to cytokines. The CD4/CD8 ratio was assessed by flow cytometry in CD3 T cells at day 7 and 14 of in vitro expansion when growth media was supplemented with different cytokines (IL‐2, IL‐7, IL‐15, and combinations thereof ). FIGURE S6. Antigen‐specific cytolysis and NFAT‐RE inducible delivery function. FRα‐specific CAR T cells manufactured using the new process induced cytolysis in (a) FRα+Luc [file BTM2-9-e10605-s002.pdf]

Supporting Information

Primary T-cell-based Delivery Platform for *in vivo* Synthesis of Engineered Proteins

Harikrishnan Radhakrishnan[1], Sherri L. Newmyer[1], Marvin A. Ssemadaali[1], Harold S. Javitz[2], Parijat Bhatnagar[1,]*

[1]Biosciences Division, [2]Education Division
333 Ravenswood Ave, SRI International, Menlo Park, CA 94025

**Parijat.Bhatnagar@sri.com*

Running title: Primary T-cell-based delivery system
DOI: 10.1002/BTM2.10605

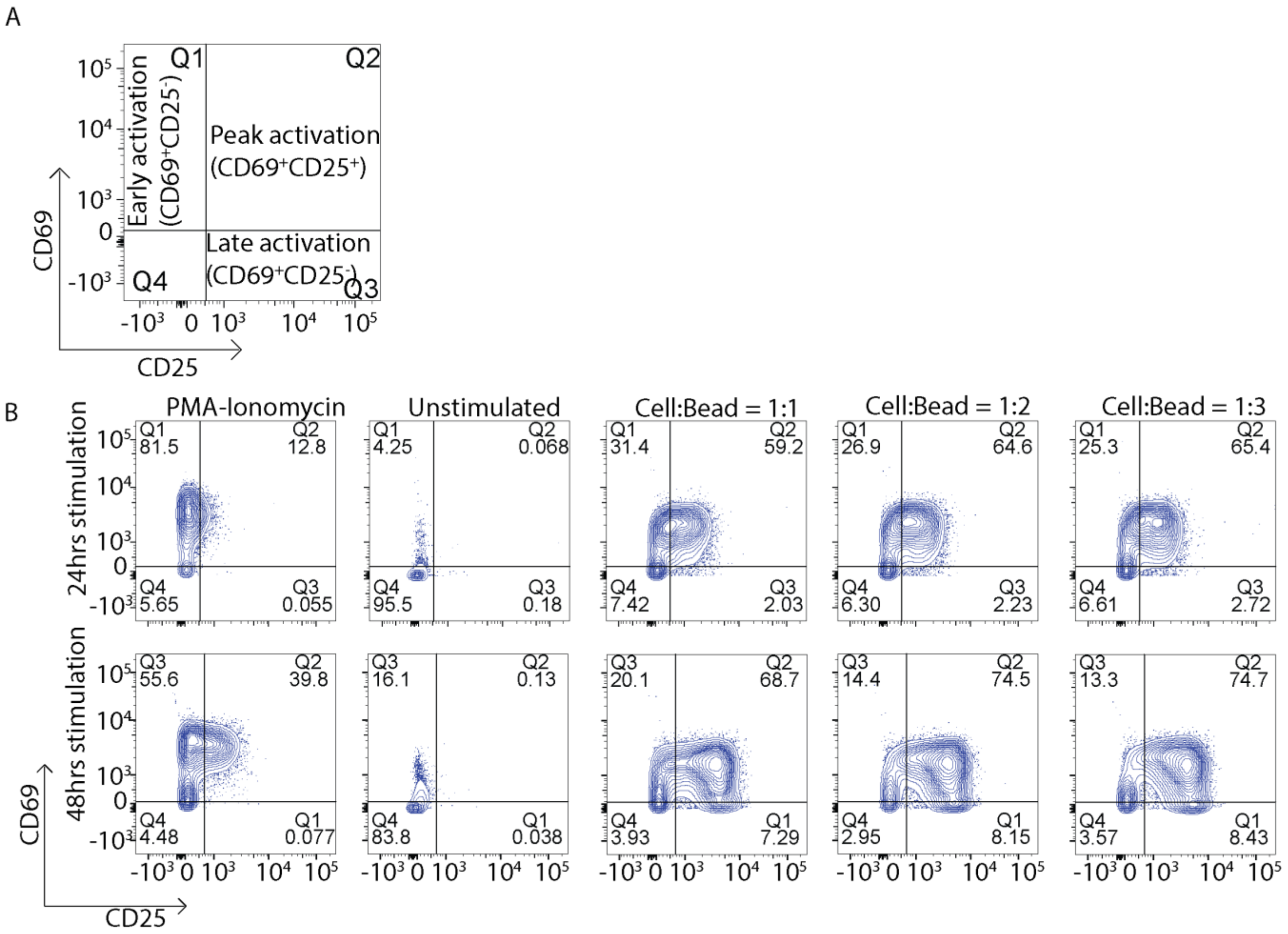

Figure S1: Strategy for evaluating CD3 T cell activation. (A) Schematic of the gating strategy used for assessing early ($CD69^{+}CD25^{-}$), peak ($CD69^{+}$CD25-) and late ($CD69^{-}CD25^{+}$) activated CD3 T cells by flow cytometry. **(B)** Representative plots showing CD69 and CD25 expression in stimulated vs. non-stimulated CD3 T cells.

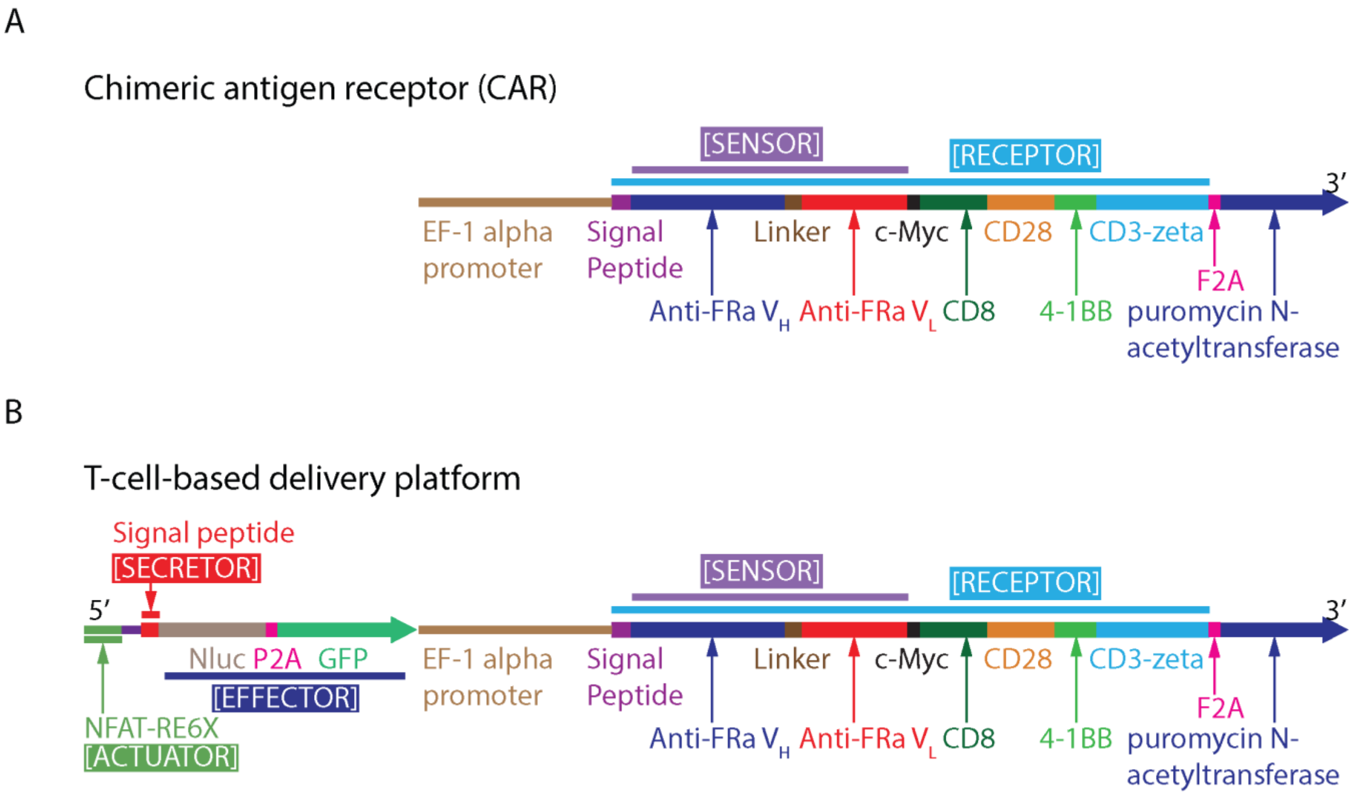

Figure S2: Schematic of the genetic payloads. (A) chimeric antigen receptor (CAR) only, 5.6 kb and **(B)** T-cell-based delivery system comprising of CAR and NFAT-RE inducible transgene, 7.2 kb.

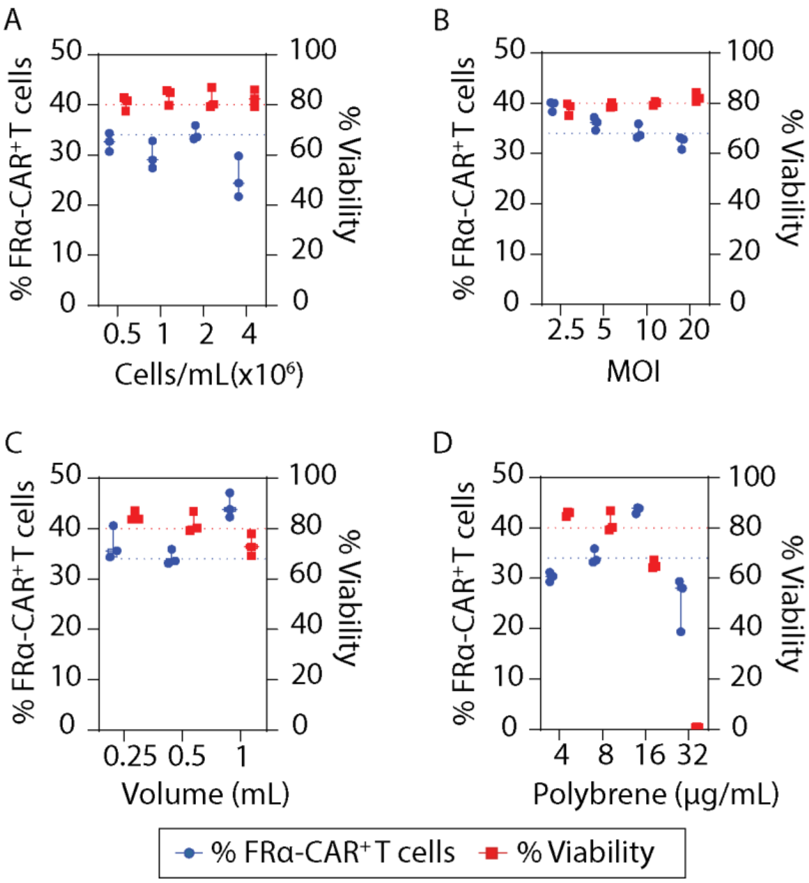

Figure S3: Additional factors affecting transduction of primary T cells with lentivectors. FRα-CAR expression (% FRα-CAR$^+$ T cells on left Y-axis) and T-cell viability (% Viability on right Y-axis) were assessed by flow cytometry after varying factors affecting transduction: **(A)** T-cell concentration in transduction reaction, **(B)** lentivector multiplicity of infection (MOI), **(C)** transduction reaction volume, and **(D)** polybrene concentration. Transduction efficiency was determined after 5 days. All results are represented as mean ± SD.

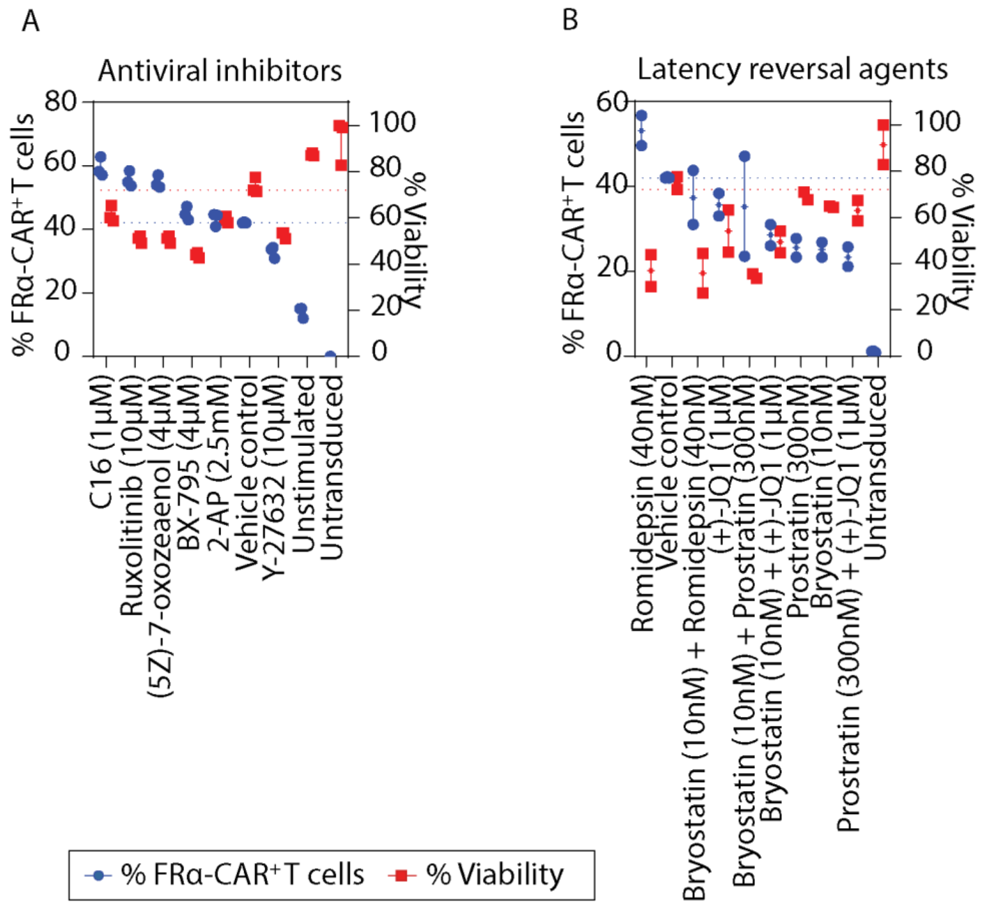

Figure S4: Exploratory screen of chemical additives for improving transduction of primary T cells with lentivectors. FRα-CAR expression (% FRα-CAR$^+$ T cells on left Y-axis) and T-cell viability (% Viability on right Y-axis) was assessed by flow cytometry after concomitant treatment with **(A)** antiviral inhibitors (AVIs), and **(B)** latency reversal agents (LRAs) during lentivector transduction. Transduction efficiency was determined after 5 days. All results are represented as mean ± SD.

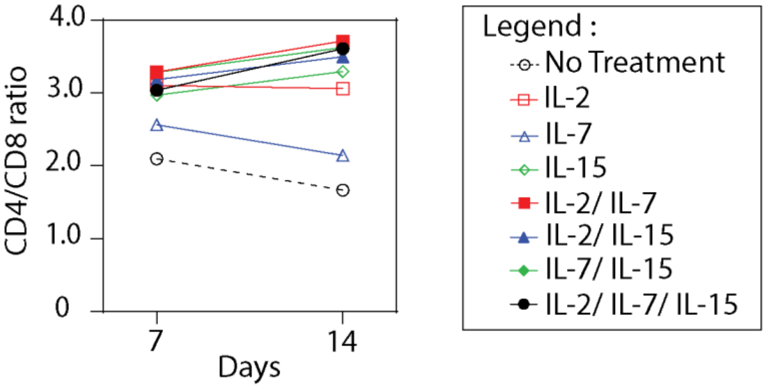

Figure S5: Change in the proportion of CD3 T cell subsets in response to cytokines. The CD4/CD8 ratio was assessed by flow cytometry in CD3 T cells at day 7 and 14 of *in vitro* expansion when growth media was supplemented with different cytokines (IL-2, IL-7, IL-15, and combinations thereof).

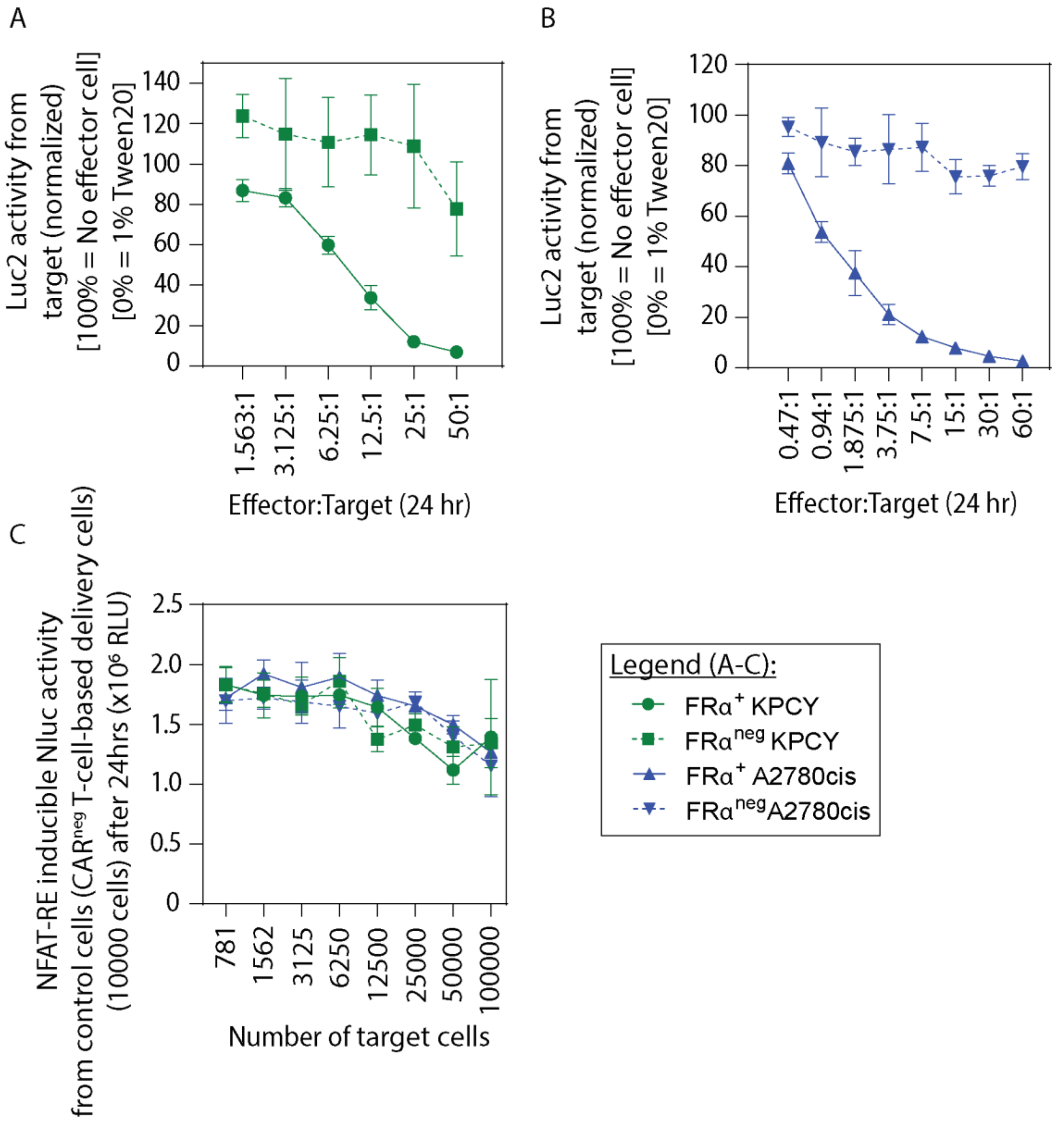

Figure S6: Antigen-specific cytolysis and NFAT-RE inducible delivery function. FRα-specific CAR T cells manufactured using the new process induced cytolysis in **(A)** FRα$^+$Luc2-2A-E2Crimson$^+$ KPCY and **(B)** FRα$^+$Luc2-2A-E2Crimson$^+$ A2780cis target cells in a dose-dependent manner, compared to their respective antigen negative cells. **(C)** Nluc activity from primary T cells engineered without CAR but with NFAT-RE inducible delivery function, when co-cultured with antigen-positive target cells for 24 hours, did not exhibit cytolytic activity compared to antigen negative cells.
